# Bone in The Endometrium: A Review

**DOI:** 10.22074/ijfs.2016.4904

**Published:** 2016-06-01

**Authors:** Sana N Khan, Monica Modi, Luis R Hoyos, Anthony N Imudia, Awoniyi O Awonuga

**Affiliations:** 1 Department of Obstetrics and Gynecology, Section of Reproductive Endocrinology and Infertility, Wayne State University School of Medicine, Detroit, USA; 2 Department of Obstetrics and Gynecology, Wayne State University School of Medicine, Detroit, USA; 3 Department of Obstetrics and Gynecology, Section of Reproductive Endocrinology and Infertility, University of South Florida, Tampa, USA

**Keywords:** Infertility, Female, Surgery, Metaplasia, Ossification

## Abstract

To provide a comprehensive review of the published literature of patients with endo- metrial bone or osseous fragments with a view to critically examine the antecedent clinical presentation, investigations and prognosis after treatment. This systematic review of the literature includes full text articles of published case re- ports and cases series from the following computerized databases: PubMed, Ovid, and Medline between 1928 and 2013. We reviewed a total of 293 patients in 155 case reports and case series. The mean ± SD age at presentation was 32.7 ± 8.9. Approximately 88% of patients had at least one prior surgical uterine evacuation relating to pregnancy termina- tion or loss at a median gestational age of 14 weeks (range of 4-41 weeks). The most common presenting symptom was infertility (56.2%). One hundred twenty- four (66.0%) of the 188 patients attempting pregnancy after treatment achieved pregnancy prior to article publication and the majority (82.3%) were spontane- ous. Spontaneous miscarriage rate remains high (43%); however, most pregnancies ended in live-birth (55%). Bone fragments in the endometrium are most commonly found after pregnancy termina- tion, present with infertility and/or irregular menses, and upon removal, patients rapidly conceive spontaneously.

## Introduction

Following the first description of bone in the endometrium by Meyer (1), several other authors have reported cases and case series relating to calcified material in the endometrium representing bone. Reports have originated from across the globe and spanned several countries and ethnicities. Although the prevalence of this entity remains unknown, the advent of newer imaging techniques such as ultrasonography meant that the presence of bone in the endometrium is being increasingly diagnosed. Nevertheless the majority of information on this subject is still from case reports and case series (2,4). 

The origin (5,6) of and the effects of bone in the endometrium on endometrial function remain a mystery. The argument centers around whether these calcified tissues are retained fetal bone from prior termination of pregnancy (7) or osseous endometrial metaplasia (6,8). The later may result from endometrial multipotent stem cell activation (9), chronic endometritis, trauma (8,10,11), heterotopia, strong endometrial estrogenic stimulation, dystrophic and metastatic calcification among others (12). Authors of previous works on the subject have suggested that the bony fragments may function as a type of intrauterine contraceptive device (13,14) thereby leading to subfertility. Indeed, the available literature have suggested that patient complaints include not only infertility but also irregular vaginal bleeding, chronic pelvic pain and even persistent vaginal discharge (10,14,15). There is a need for a clear description of the demographic information of patients who present with this condition, specifically their antecedent pregnancy information, the complaints with which they present, and the duration of time the condition has persisted before diagnosis. Accordingly, our objectives are: first, to provide a comprehensive review of the published literature with a view to gleaning the epidemiological characteristic of patient found to have bone on radiological imaging. Second, we describe the possible pathogenesis of the condition. Third, we evaluate the different published treatment modalities for this condition and their outcome. 

## Materials and Methods

In this systematic review of the literature, we performed a comprehensive literature search on PubMed, Ovid, and Medline using medical subject headings (MESH) words including endometrium, fetal, bone, osseous fragments, pelvic imaging (Xray and ultrasound) and osseous metaplasia and all combination of the aforementioned words. All articles referenced in searched articles were also reviewed for cases missed from the original search. Only full text English, Spanish, Portuguese, Italian, French, and Turkish articles were included. One article in German (16), one in Danish (17), and one Hebrew (18) were excluded, as we were unable to find translators for these articles. Data from all articles published in French describing this phenomenon in 1928 (19) and the latest in 2013 (20) were included. One article (21) with 15 cases in which individual case information was not given were excluded for the purpose of this review. Published case series were included when individual case information was provided (6,10,22,24). 

Analyses of cases were done with respect to i. Demographic variables, ii. Antecedent pregnancies, iii. Diagnostic modalities used, iv. Pathologic examination of endometrial curetting or resection, and v. Outcomes after treatment. Demographic variables included were age, gravidity, parity and race/ethnicity. Presenting complaint(s), history of pelvic infections or pelvic inflammatory disease, menstrual symptoms, and assessment of tubal patency when performed were recorded. Antecedent pregnancy information included number of prior terminations or losses, whether terminations were medical or surgical, greatest gestational age at prior pregnancy loss or termination when multiple losses existed, and duration between such termination/loss and presentation to care. Information regarding diagnosis and treatment included how diagnosis was made, and whether a previous evaluation for their complaint(s) had been normal. This information was obtained to assess whether certain imaging modalities were less effective in diagnosis, and also to help in constructing a timeline of events between terminations or losses and final diagnosis and treatment. Also recorded information indicated the type of treatment each patient received to remove the bone fragments. Pathologic information was also collected where available and included number of bone fragments removed, lengths of bone fragments (cm), and finally a histologic examination including presence or absence of inflammation and marrow formation. Outcome data included patients’ complaints (when symptoms were reported) resolved, the proportion of pregnancies, and their outcome in those that presented with infertility. 

Continuous data were summarized using mean (SD) for age, and gestational age at delivery, while median (range) was used for gravidity and parity. Percentages were calculated for categorical data. As this was an analysis of published cases, institutional review board (IRB) approval was not necessary for this study. 

## Results

A total of 293 patients with endometrial bone or osseous materials were analyzed from a total of 155 articles. Of the factors analyzed, only patient’s age was reported in all of the 293 cases. The mean (SD) age for the cohort was 32.7 ± 8.9 years (range of 15-73 years). Similarly, 209 (71.3%) cases contained information regarding gravity and parity with a median gravity and parity of 2 (range of 0-12) and 1 (range of 0-10), respectively. Of 218 (74.4%) patients in which ethnicity was reported, the majority (65, 29.8%) were Hispanic/ Latino, while other ethnicities were less common: 52 (23.8%) were Black or of African descent; 39 (17.9%) were Caucasian from Europe and Australia, while 13 (6%) were Caucasian from North America; 18 (8.3%) were Asian/Pacific Islander, 16 (7.3%) were from the Indian Subcontinent, while 14 (6.4%) were of Middle Eastern (including Turkey) ethnicity; and the least common reported ethnicity in 1 case [0.5, 95% confidence interval (CI): 0.02-2.9%] was Native American. The United States of America (USA) and France reported 21 articles each (13.6%), followed in descending order by; the United Kingdom (UK) (12.3%), Turkey (6.5%), Italy and India (5.8% each), Brazil and Canada (3.9% each), Netherlands and Chile (3.2% each), China and Mexico (2.6% each), Romanian and Venezuela (1.9% each), Pakistan, Greece, Spain, Hong Kong, Sweden, Columbia and Tunisia (1.3% each), while Korea, Ireland, Australia, Ivory Coast, Jamaica, Japan, Jordan, Germany, Ghana, Qatar, Morocco, Israel, Algeria, Democratic Republic of Congo and Denmark all had one publication relating to bone in the endometrial in our study (0.6% each). It is noteworthy that majority of the cases were reported by large cases series from Brazil (6), Korea (22) and the UK (10). The majority of the patients were being investigated for infertility which was reported in 150 cases out of a total of 267 (56.2%) patients in whom the absence or presence of presenting symptoms were reported. Of the 170 patients in whom the menstrual history was reported or can be deduced from the report, only 53 (31.2%) reported regular menses, 105 (61.8%) reported irregular menses, while 12 (7.1%) were postmenopausal. Other presenting symptoms are reported in Table 1. 

**Table 1:** Presenting symptoms


Symptom	Frequency (%)

Infertility	150/267 (56.2%)
Irregular bleeding	53/267 (19.8%)
Vaginal discharge	17/267 (6.4%)
Dysmenorrhea	7/267 (2.6%)
Dyspareunia	3/267 (1.1%)
Pelvic pain	21/267 (7.9%)
Recurrent pregnancy loss	1/267 (0.4%)
Asymptomatic	15/267 (5.6%)


The median number of prior terminations or losses was 1 (range of 0-6). In other words, the vast majority of the 239 patients in whom number of pregnancy terminations or losses was reported, 175 (73.2%) had only 1 preceding termination or loss, 29 (12.1%) had 2 terminations or losses and 25 (10.5%) had 3 or greater. Of note, 10 cases (4.2%) reported no prior pregnancy termination or loss. Two hundred forty-four cases reported on whether termination or loss was spontaneous or surgically accomplished. Of these, twenty-nine cases (11.9%) reported spontaneous losses with no surgical intervention, while the remaining 215 cases (88.1%) reported surgical terminations or curettage. The median gestational age of preceding pregnancy was 14 weeks (range of 4-41 weeks). The median duration between last pregnancy and presentation to care or incidental diagnosis was 5 years (range of 1-40 years). Diagnostic modalities used to diagnose the presence of endometrial bone or osseous materials are shown in Table 2.

**Table 2:** Different diagnostic tests used for the diagnosis of endometrial bone


Diagnostic test	Frequency (%)

Ultrasound	161/246 (65.4%)
Dilation and curettage	38/246 (15.4%)
Radiography	1/246 (0.4%)
HSG	27/246 (11.0%)
Hysteroscopy	3/246 (1.2%)
Visualization on exam	8/246 (3.3%)
Endometrial biopsy	8/246 (3.3%)


HSG; Hysterosalpingogram.

**Fig.1:**
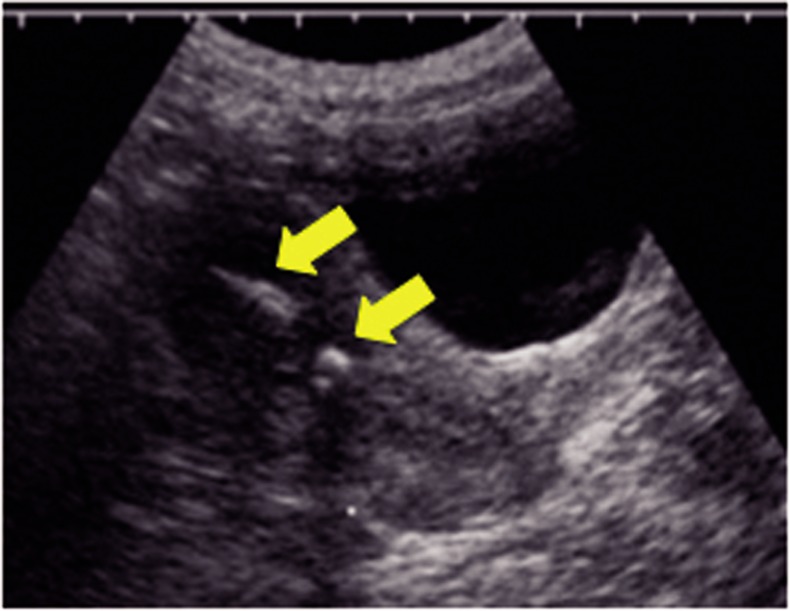
Bone fragments within the endometrium visualized sonographically. Arrows point to bone fragments.

Interestingly, the initial diagnostic method was reported as normal in 11 of 123 (8.9%) cases that had ultrasound as the initial imaging modal- ity. Figure 1 demonstrates an ultrasound image of bone in the endometrial cavity. Another interesting finding was the presence of uterine anomalies in the cohort. Of all cases reviewed, 4 (1.4, 95% CI: 0.4-3.7%) patients were noted to have Müllerian duct anomalies (MDA) including: 1 uterine didel- phys and 3 septate uteri. Of the patients evaluated for infertility, assessment of tubal patency was re- ported in 82 cases. Of these, 61 (74.4%) had bi- lateral tubal patency, 5 (6.1%) had unilateral tubal patency, while 16 (19.5%) had bilateral tubal oc- clusion. History of previous pelvic inflammatory disease was rarely reported.

The presence or absence of such a history was reported in only 15 case reports, of which only 6 (40%) had a positive previous history. Endometrial location of the bone fragment was recorded in 33 cases (11.3%) (Table 3). 

**Table 3:** Location of bone fragments within endometrium and/or endometrial cavity

Location of fragments	Frequency (%)	95% CI
Posterior	27/67 (40.3%)	28.6-52.0%
Intracavitary	19/67 (28.3%)	17.6-39.2%
Anterior	8/67 (11.9%)	4.2-19.7%
Multiple surfaces	13/67 (19.4%)	9.9-28.9%

CI; Confidence interval.

**Fig.2:**
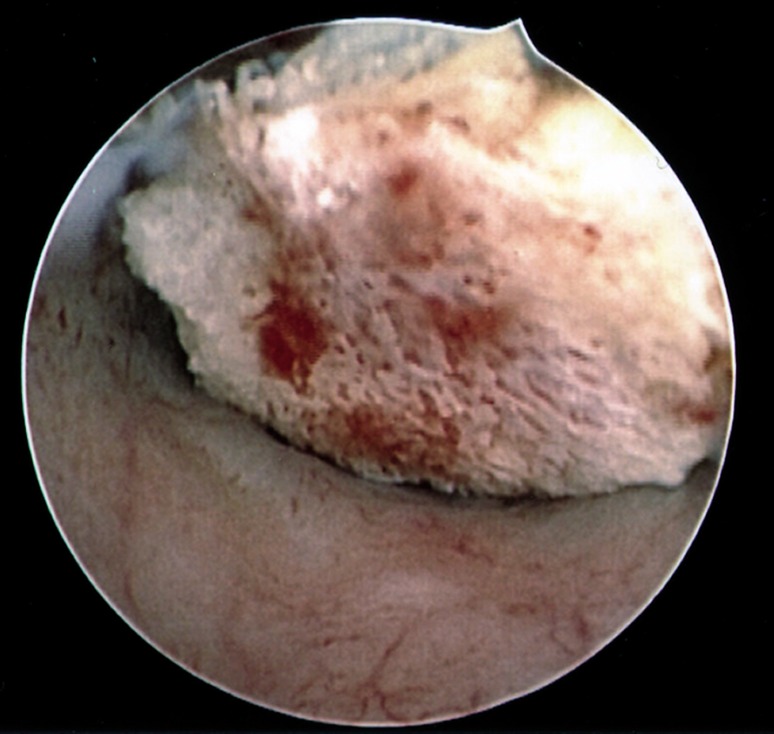
Image of bone in endometrium obtained hysteroscopically prior to removal. Histopathologic examination was consistent with bone fragments.

Two hundred seventy cases (92.2%) included in- formation about treatment, of which the most com- mon treatment modality for removal of bone frag- ments was hysteroscopic based excision in 182 cases (67.4%), whereas non-hysteroscopic treat- ments (biopsy, forceps, dilation and curettage, and hysterectomy) occurred in 87 (32.2%) patients. Figure 2 shows calcified tissue located within the endometrium, which was pathologically deter- mined to be bone. It is noteworthy that in 61.2%, the bony fragment involved the posterior uterine wall. Most patients had 4 or more bony fragment (58.6%) with 68.9% of these measuring 1 cm or greater. Two hundred eighty-nine (98.6%) cases reported pathologic evaluation and all (100%) detected the presence of bone. In two cases, frag- ments were not removed; therefore, pathologic confirmation was never obtained. Of the 289 cases reporting pathology, only 17 (5.9%) were noted to have marrow formation and 11 (3.8%) were noted to have cartilage. Of the 289 cases, the presence or absence of inflammation in the retrieved endo- metrial specimen was reported in 224 (76.5%), of which 151 (67.4%) showed evidence of inflamma- tion or infection.

Symptom resolution was reported in 52 of 64 (81.3%) patients who did not present with infer- tility. Symptom non-resolution was reported in 3 (4.7%), while 9 patients (14.1%) were lost to fol- low up. One hundred and eighty-eight (64.2%) cases reported on whether patients did or did not attempt to get pregnant after treatment and of these, 124 (66.0%) attempted to get pregnant. Of these, 90 (72.6%) achieved pregnancy prior to article publication, 16 (12.9%) reported persis- tent infertility, while 3 (2.4%) stopped attempting pregnancy for reasons including age, decreased ovarian reserve and tubal non-patency. In the re- maining 15 patients (12.1%), the outcome of fer- tility was not reported. Of the 90 (72.6%) patients who achieved pregnancy, 74 (82.2%) achieved pregnancy spontaneously, while 10 (11.1%) need- ed infertility treatment or assisted reproductive technology (ART), whereas in 6 cases (6.7%), how pregnancy was achieved was not specified. Of the 90 patients who achieved pregnancy, information on duration of attempt prior to success was provided in 25 cases. The majority of patients who were attempting to achieve pregnancy after treatment conceived within 3 months (36%), followed by 2 months (16%) with durations from 1 to 9 months making up the remainder. A paucity of information was reported regarding pregnancy outcomes. 

Thirty cases reported the outcomes of pregnancies after treatment of endometrial bone. Of those, 17 resulted in live births with only one preterm case, which was a twin pregnancy. Of the remaining 13, 11 were spontaneous abortions and 2 were ectopic pregnancies (Table 4). 

**Table 4:** Pregnancy outcomes


Pregnancy outcome	Frequency (%)	95% CI

Spontaneous pregnancy	74/90 (82.2%)	72.9 to 88.9%
Pregnancy with infertility treatment	10/90 (11.1%)	5.9-19.4%
Pregnancy (unspecified if spontaneous of with infertility treatment	6/90 (6.7%)	2.8-14.1%
Full term birth	16/30 (53.3%)	36.1-69.7%
Preterm birth	1/30 (3.3%)	<0.01-18.1%
Ectopic	2/30 (6.6%)	0.8-22.4%
Spontaneous abortion	11/30 (36.6%)	21.8-59.8%


CI; Confidence interval.

## Discussion

A clearer characterization of patients with bone in the endometrium has emerged from this com- prehensive review of published cases reports and case series. Most reports were of patients of South American, North American/European, or African descent; however, the majority of published re- ports originated from the UK and the USA. Most patients who were in their earlier thirties reported having undergone at least 1 pregnancy termina- tion or loss often in the early second trimester. Graham et al. (10) from the UK reported on 11 West African women who underwent termination of pregnancies (TOPs) in their countries of origin and presented with infertility related to retained intrauterine bone. Termination of pregnancies in developed countries is often illegal; therefore, such operations are more likely to be performed by inexperienced practitioners and those with no medical qualifications. Due to associated social stigma in these communities, TOPs tend to be per- formed much later than usual, carrying a greater risk of being incomplete. It, therefore, seems that the bone or osseous materials are more likely of fetal origin related to such TOPs. The USA and the UK have a large population of immigrants; there- fore, it is tempting to speculate that in the major- ity of cases, sub-optimal management in under- developed countries prior to immigration might be responsible for this disorder. The prevalence of en- dometrial fetal bone following TOPs is unknown. However, ultrasounds are increasingly being used in the western world to evaluate patients with in- fertility, uterine fibroids, other pelvic masses and cancer, endometriosis and pelvic pain; therefore, the presence of endometrial bone is more likely to be identified. The high number of reported cases in the western world may be because gynecologists in the USA and the UK are more willing to subject patients to pelvic ultrasound, and are more likely to publish such cases when encountered.

A major point of debate regarding this topic is the pathophysiology of bone in the endometrium. The prevailing hypothesis posits that these bone fragments are retained fetal bone fragments left embedded in the endometrium (13-15) following uterine evacuation after pregnancy termination or a miscarriage. 

The reasons for the findings of endometrial os- seous material in the 9 reported nulligravidas and those with early first trimester terminations or loss when fetal bone formation is not known to occur are unknown. Endometrial bony fragments in these cases may be due to metaplasia of the stromal cells of the endometrium into osteoblastic cells that ma- ture to produce bone (25-27). In support of this theory, 16 articles in the current series reported the presence of marrow formation on pathologic evaluation of the retrieved osseous fragments. It noteworthy that the reported gestational age at time of loss or termination in these cases was less than 20 weeks and as early as 10 weeks, which is prior to when fetal medullary hematopoiesis is known to occur. In addition, multiple groups have confirmed the presence of multipotent cells in the endometrium. It is known that endometrial stem cells can differentiate into bony material (9, 28). Consistent with this, metaplasia of the endometrial stromal cells (usually fibroblasts) into osteoblasts has been proposed as a bone-deriving mechanism (29) . Moreover, Parente et al. (6) and Cayuela et al. (30) performed genetic analysis on the bony fragments retrieved from endometrial curetting (8 of 14 cases and 1 case, respectively) and found them to be genetically identical to cells from the mother in all cases analyzed. These findings provide support to the possibility that some endometrial osseous material might come either from maternal endometrial stem cells or from metaplasia of maternal stromal cells. Collectively, these suggest that bone fragments in some of these patients may not be of fetal but of maternal origin. 

Several studies have tried to relate the pathophysiology of the condition with the presenting symptoms. Over 70% of patients presented with irregular menstrual periods and/or infertility; however, the range of presenting complaints also included pelvic pain and vaginal discharge, while a small minority was asymptomatic. Abnormal menstruation in patients with retained bony fragment(s) may be due to higher prostacyclin concentrations noted in their menstrual fluid, which is known to cause vasodilation and increased uterine bleeding. Also it was proposed that the physical presence of ossified intrauterine material may cause uterine irritation and that the ensuing pelvic pain may result from associated increased prostaglandins levels (15). However, in the present study, we found that <2% of patients with retained endometrial bone presented with dyspareunia and pelvic pain. The presence of osseous material within the endometrial lining or in the uterine cavity (10,29) may act in a similar manner to a non-hormonal intrauterine contraceptive device (13) by increasing menstrual fluid prostaglandin and prostacyclin (15) or by causing chronic endometritis (CE)-like reaction (31). Indeed, over 52% of the patients who had their endometrial curettings subjected to histopathological examination had evidence of inflammation. Interestingly, despite the fact that all patients had bone in the endometrium, the presence or absence of pelvic inflammatory disease was only reported in 15 patients and of these, only 6 (40%) reported a history of prior infections causing pelvic inflammatory disease. Reasons for these findings may include underreporting in the case reports as well as a failure to elicit this history from patients. One theory is that bone in the endometrium acts as a foreign body (10), which can then be a nidus for infection. However, based on the available evidence, it appears that patients with bone in endometrium do not have an increased propensity for pelvic inflammatory disease. 

The most common method of diagnosis was ultrasound; the use of other modalities such as hysterosalpingography (HSG) (23) may represent earlier time period when sonography was not as widespread. Interestingly, there were several reports of prior ultrasound evaluation (8.9% in this series) and HSG that were reported as normal prior to the definitive diagnosis. These would suggest that either the prior studies were inadequate or that the process through which endometrial bone ensues is chronic in nature as with osseous metaplasia. Unfortunately, our numbers are too small to make a clear assessment of this finding. 

One very interesting finding in our study was the presence of MDA in the cohort. Of all cases reviewed, there were 4 cases of MDA with one uterine didelphys and three septate uteri. This finding has been previously noted as well as the importance of characterizing the anomaly prior to any instrumentation (32). The question of whether patients with uterine anomalies are more predisposed to retention or development of bone in the endometrial cavity was suggested by Chervenak et al. (33). To address this question, we analyzed the frequency in our cohort and compared this with frequency data from other populations. In a large case series published in 2008, Saravelos et al. (34) determined the frequency of uterine anomalies in the general population to be 6.7%, in the infertile population to be 7.3% and in the recurrent miscarriage population to be 16.7%. In our cohort, the frequency was much less than any of the previously described populations at 4/293(1.4%), suggesting that there is not an increased risk of the finding of bone in the endometrium in patients with uterine anomalies. 

Most treatments were hysteroscopy based (68%), as it is the most appropriate method to remove intrauterine pathology because it is less invasive, more efficacious and less costly than other options (35). Of the other 32% treated cases, the most common treatment was dilation and curettage, which may be a function of the time period prior to the widespread use of hysteroscopy. Although the first description of hysteroscopy was published in the 1920s (36), widespread use of this technique did not appear until the mid-1970s (37). Of note, 29 cases in our series were published prior to 1980. Interestingly, 12 cases reported hysterectomy for the treatment of bone in the endometrium. Most of these cases described patients who were perimenopausal and who had completed childbearing. However, at least three patients from different articles were in their early-mid 20s and presented with complaints of infertility but underwent hysterectomy; the exact indication(s) for such an operation in these young women was not stated in these reports. 

Perhaps the most important finding of the present study was that the majority of patients with this condition were able to become pregnant spontaneously after treatment. Furthermore, most patients who did conceive were able to do so quickly, indicating that the majority conceived in less than 6 months after treatment. Additionally, in over 50% of these pregnancies, the outcomes were live births. The rate of spontaneous miscarriage of 36% is higher than even the most robust incidence estimates, raising concern for remnants of osseous material still retained in the endometrium (38,39). Additionally, for those patients who did not desire pregnancy, the majority did experience symptom relief after treatment. 

The purpose of this study is to characterize these patients, increase awareness that the condition exists, and provide guidance on how to counsel patients regarding prognosis after treatment when this rare problem is encountered. The information therein is intended for use by practitioners when they encounter this problem, because most will never encounter a case judging by the rarity of the condition. Given that most cases resulted from uterine evacuation in the second trimester, surgical uterine evacuation under ultrasound guidance at this stage of pregnancy may help and perhaps should be routinely used in these cases. However, there is no scientific evidence for such a recommendation as retained products conception could still be encountered despite concomitant use of ultrasound (40). 

There are several strengths to this study, the most important one being the number of cases included for analysis, which to our knowledge is the largest in the literature. Furthermore, we included cases reported in all languages except 5 case reports that were written in languages in which we were unable to obtain help in translation; therefore, our list cannot be regarded as complete. 

We wish to emphasize several limitations associated with this study. This is a synopsis of case reports and case series often with different emphasis and heterogeneity on facts presented; however, the study design is necessary for studying very rare conditions such as bone in the endometrium. Retrospective epidemiologic studies are susceptible to being biased, and data entries into patient’s health record are uncontrolled or unsupervised often with several important missing data as shown in this study. Furthermore, the bias to describe favorable as opposed to unfavorable outcomes is well documented and may contribute to the success rate of conception after treatment. Therefore, caution is needed to interpret the data presented here. By its nature, it was impossible to estimate the prevalence of this condition in the general public as this is not a cohort or prospective study. Also, the relatively small number of cases reported may make the conclusions resulting from sub-analysis flawed. In addition, as cases went as far back as 1928 (19), it is unclear how accurate cases reported in early years were, because it was only during the 1960s that the first medical applications of ultrasound were being tested and it was not until the 1970s that the technology became widely available (41). 

## Conclusion

The present study is the largest evaluation of patients with findings of bone in the endometrium. We describe the most common demographic information as well as patients’ presentation and sequel after treatment. Based on available research, it appears that the pathogenesis of this condition involves at least some component of osseous metaplasia; however, further studies are warranted to better understand the pathophysiology of this condition. 
